# Surveillance of human leptospirosis infections in Ukraine between 2018 and 2023

**DOI:** 10.3389/fpubh.2024.1394781

**Published:** 2024-06-12

**Authors:** Pavlo Petakh, Viktoriia Tymchyk, Oleksandr Kamyshnyi

**Affiliations:** ^1^Department of Biochemistry and Pharmacology, Uzhhorod National University, Uzhhorod, Ukraine; ^2^Department of Microbiology, Virology, and Immunology, I. Horbachevsky Ternopil National Medical University, Ternopil, Ukraine; ^3^Transcarpathian Regional Center for Disease Control and Prevention, Uzhhorod, Ukraine

**Keywords:** leptospirosis, zoonoses, *Leptospira interrogans*, epidemiology, Ukraine

## Abstract

Leptospirosis is a bacterial disease that affects both humans and animals worldwide. Currently, a positional war is ongoing in Ukraine, and the military is encountering a significant number of rodents in trenches and dugouts, which are known reservoirs for *Leptospira*, the causative agent of leptospirosis—a potentially dangerous infectious disease with a high mortality rate. The civilian population is also at potential risk of leptospirosis. The destruction of the Kakhovka Dam on June 6, 2023, has led to widespread devastation and human suffering. In the short term, there is a significant risk of rodent-borne diseases such as leptospirosis. We utilized data from the Ukrainian Centre for Disease Prevention Control and observed a substantial increase in prevalence in 2023. The notification rate in Ukraine in 2023 was 1.06 per 100,000 persons, which is higher than that of other countries in the European Union. Particular attention is being given to Zakarpattia Oblast, located on the western border of Ukraine, which shares boundaries with Romania, Hungary, Poland, and Slovakia, with an extremely high incidence rate of 12.08 per 100,000 persons. Based on these findings, we recommend education and awareness campaigns, vaccination, personal protective measures, and improved surveillance to address the increasing incidence of leptospirosis in Ukraine.

## 1 Introduction

Leptospirosis is a global zoonotic disease caused by spirochaetes of the genus *Leptospira* (1). Leptospires are currently classified into subclade P1 (formerly pathogenic), P2 (formerly intermediate), S1 (formerly described as the saprophyte group), and S2 (a new subclade that includes *L. idonii*) (2). Leptospires, which reside in the kidneys of various mammals, are excreted through urine and primarily spread indirectly through environmental contact (3). This disease thrives in warm, humid environments, making it particularly common in wet tropical and subtropical regions (4). A 2015 systematic review estimated that there are approximately one million cases of leptospirosis and approximately 60,000 related deaths annually worldwide (5).

Most cases are confirmed through serologic testing, such as the microscopic agglutination test (MAT) or IgM enzyme-linked immunosorbent assay (ELISA), during the acute phase (4). Polymerase chain reaction (PCR) can detect the pathogen's nucleic acid in blood, urine, or cerebrospinal fluid during the acute phase, but culturing is slow and less sensitive (6). Timely antimicrobial treatment can mitigate severity and duration, yet underreporting is likely due to mild cases, unspecific symptoms, and challenging laboratory findings.

The ongoing conflict in Ukraine poses a unique occupational risk to military personnel (7), who have an increased risk of infection due to exposure to contaminated water sources and potential reservoir hosts such as rodents (8). Studying the incidence of leptospirosis among servicepeople of the Armed Forces of Ukraine in 2022, Ogorodniychuk et al. reported that 6 contract servicepeople and 40 mobilized military personnel contracted leptospirosis, accounting for 32% of all cases (9). The civilian population is at risk after the destruction of the Kakhovka Dam in 2023, leading to potential outbreaks of rodent-borne diseases, including leptospirosis and tularemia (10).

The objective of this study is to report on the human leptospirosis cases in Ukraine from 2018 to 2023, examining the data at national and regional levels and across different age groups. The study also compared the findings with those of countries in the European Union (EU).

## 2 Materials and methods

Data on the incidence of leptospirosis were extracted from the database of the Ukrainian Centre for Disease Prevention Control (CDC).

### 2.1 Surveillance system

Leptospirosis in Ukraine is a disease subject to mandatory registration in accordance with the order of the Ministry of Health of Ukraine dated July 30, 2020, No. 1726. Each case of leptospirosis is reported to the local epidemiological departments of the CDC within 2 h of detection via telephone communication. An emergency notification about an infectious disease is then drafted, with a paper copy transferred within 18 h, as regulated by the Ministry of Health of Ukraine through order No. 1 dated January 10, 2006.

All medical personnel, no matter the type of organization they work for, as well as employees of other medical services who obtain information about an individual's health status during their duties, are responsible for completing an emergency notification about an infectious disease.

### 2.2 Diagnosis

#### 2.2.1 Epidemiological history and clinical data

Medical personnel obtain detailed information regarding the patient's history, including exposure to potential sources of leptospirosis, such as contaminated water, contact with animals, and recent travel. The clinical criteria for diagnosing leptospirosis, as determined by the order of the Ministry of Health of Ukraine No. 905 of December 28, 2015, include fever or at least two of the following symptoms: chills, headache, myalgia, hyperemia of the conjunctiva, hemorrhages in the skin, and mucous membranes, rash, jaundice, myocarditis, meningitis, kidney failure, and respiratory manifestations such as hemoptysis. However, clinical and epidemiological data alone are not sufficient for confirming the diagnosis. For confirmation, cases are tested using PCR or MAT.

#### 2.2.2 Laboratory testing

Suspected cases among humans were identified in local healthcare facilities based on clinical symptoms and histories of potential animal exposure. Blood samples from patients suspected of having leptospirosis were collected and analyzed using the MAT test at the Especially Dangerous Infections (EDI) laboratories. Initially, paired blood samples were tested at dilutions of 1:5 and 1:50. If a positive reaction occurred at these titres, further dilutions were performed at 1:10, 1:100, 1:200, and beyond, following the protocol outlined in the Methodological Recommendations 9.1.1.098-02 for Anti-Epidemic Measures and Laboratory Diagnostics of Leptospirosis, approved by the Decree of the Chief State Sanitary Doctor of Ukraine No. 39, dated December 11, 2002 (11). To validate the results, a control culture diluted 1:2 in phosphate-buffered saline was utilized. The endpoint is defined as the dilution of serum that shows 50% agglutination, leaving 50% free cells compared with this control culture, as described in the World Organisation for Animal Health (WOAH) Leptospirosis Terrestrial Manual (12). The results of the test are reported as the endpoint dilution of serum or as a titer, which is the reciprocal of the endpoint serum dilution. Antibody titers of ≥1:100, combined with clinical and epidemiological data, were considered indicative of infection (13). A fourfold increase in antibody titres between paired samples provided strong evidence of acute infection. MAT testing was performed using 14 *Leptospira* spp. serovars (Supplementary Table S1) in accordance with local protocols (11).

It is important to note that MAT was used for convalescent sera rather than during the acute phase (14). In patients who died with a clinical diagnosis of leptospirosis, the disease was confirmed through PCR analysis of sectioned kidney tissue, using PCR primers based on the *lipL32* gene (15).

### 2.3 Reporting

The reporting mechanism was structured to ensure accurate and timely dissemination of information to relevant stakeholders. The following steps were involved in the reporting process:

#### 2.3.1 Notification rate

Notification rates are broken down by region and age group. The age groups are defined as follows: children are those aged 0–17 years, while adults are those aged 18 years and older.

The analysis included 22 regions of Ukraine and the capital city, Kyiv. The Autonomous Republic of Crimea, the city of Sevastopol, and the Luhansk and Donetsk regions were excluded due to the lack of relevant data resulting from their temporary occupation.

For the regions included in the analysis, mean annual notification rate data from 2018 to 2023 were utilized to calculate percentiles. The classification was as follows: low notification rate (less than the 25th percentile): <0.292 per 100,000; moderate notification rate (between the 25th and 75th percentiles): an average of 0.292 and 1.056 per 100,000; and high notification rate (greater than the 75th percentile): >1.056 per 100,000.

#### 2.3.2 Timely reporting

Healthcare providers were required to promptly report suspected or confirmed cases to the CDC within 18 h.

## 3 Results

### 3.1 Notification rate

**Figure 1:**
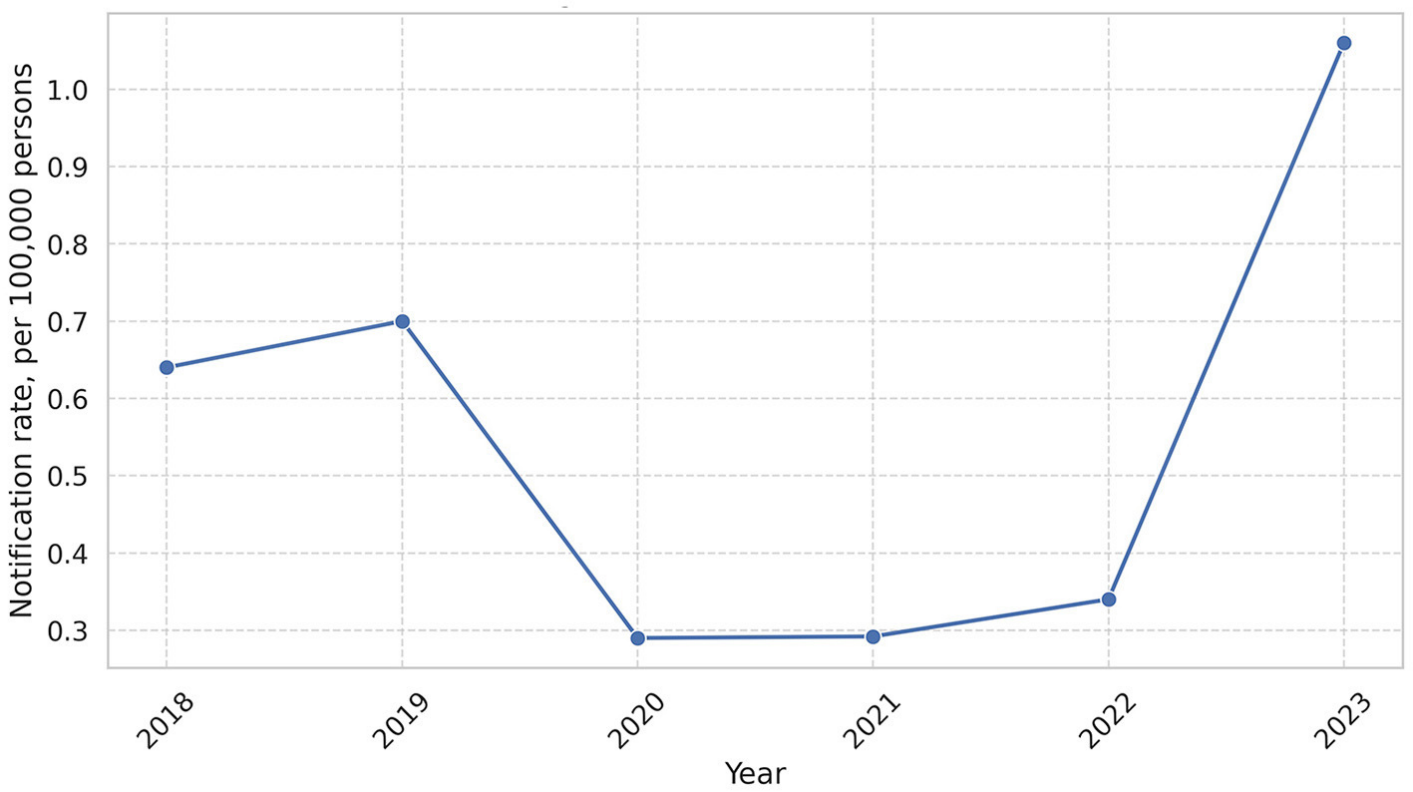
Leptospirosis notification rate in the period 2018–2023. In this figure, we can observe peak of leptospirosis in 2023 (1.06 per 100,000).

We analyzed the total notification rate in Ukraine for the period from 2018 to 2023. Over the six-year period, a cumulative total of 1,384 leptospirosis cases were reported in Ukraine. The annual distribution of cases is as follows: 273 cases (0.64 per 100,000) in 2018, 295 cases (0.70 per 100,000) in 2019, 120 cases (0.29 per 100,000) in 2020, 122 cases (0.29 per 100,000) in 2021, 141 cases (0.34 per 100,000) in 2022, and 433 cases (1.056 per 100,000) in 2023. In 2023, Zakarpattia reported the highest absolute number of cases, with 150, which accounted for 34.6% of all leptospirosis cases in Ukraine (Figure 1, Supplementary Table S2).

### 3.2 Regional distribution

**Figure 2:**
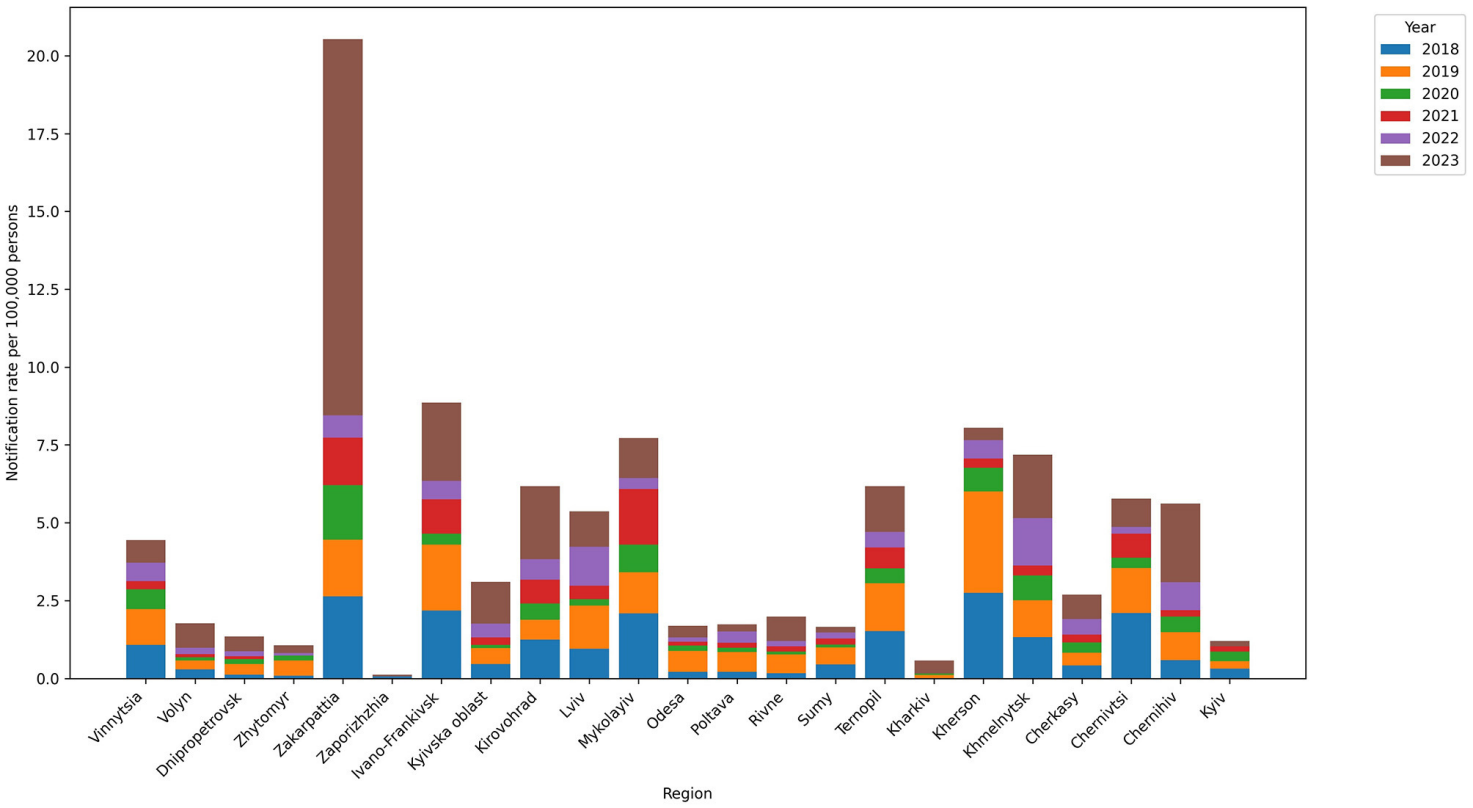
Leptospirosis notification rate in regions in the 2018–2023 period. The highest notification rates were observed for Zakarpattia (3.42), Ivano-Frankivsk (1.48), Mykolayiv (1.34), Kherson (1.29), and Khmelnytskyi (1.2).

**Figure 3:**
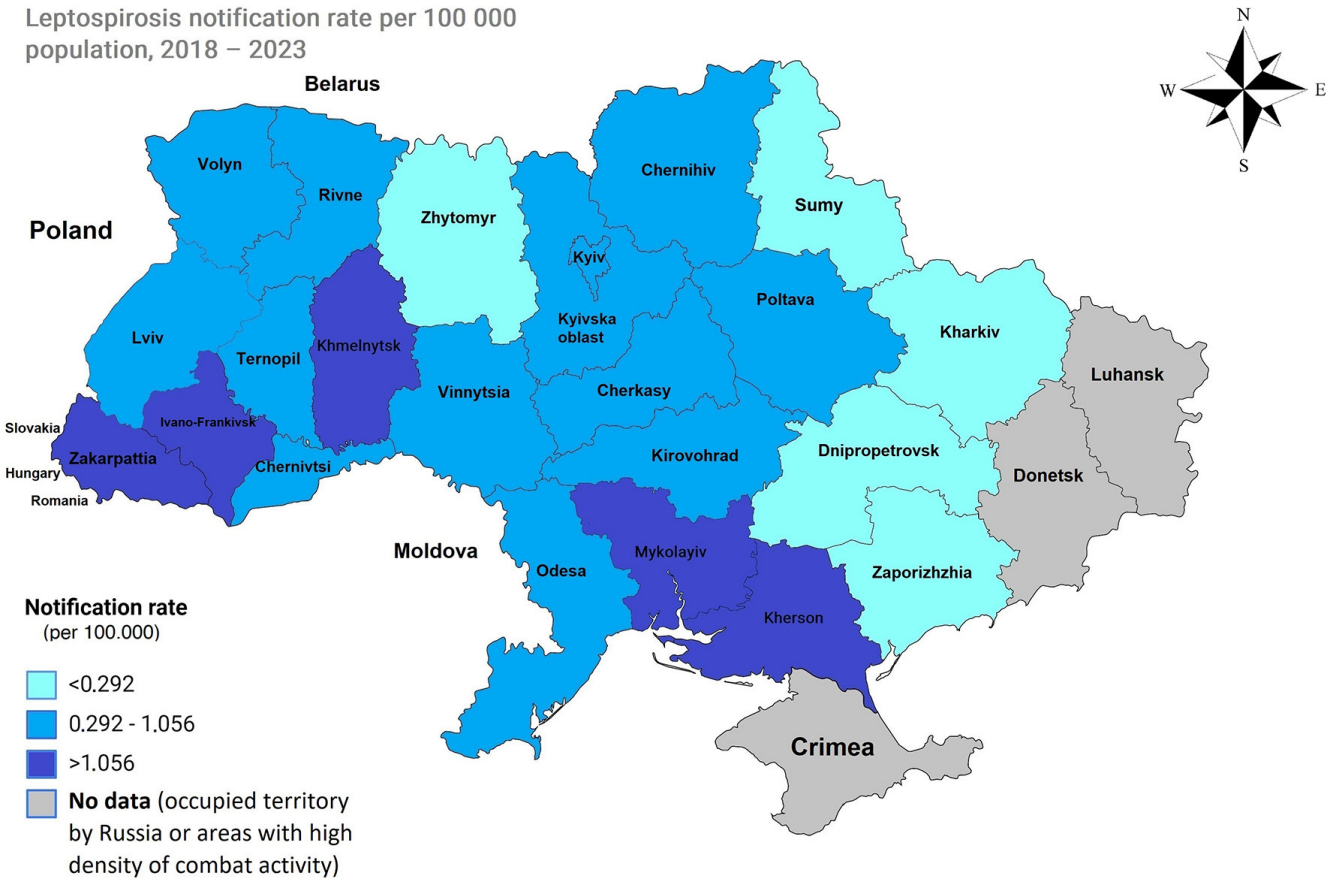
Map of Ukraine with average notification rates in the period of 2018–2023. Low incidence (less than the 25th percentile), 0.292 per 100,000, is represented by regions displayed in light blue. A moderate incidence (between the 25th and 75th percentiles), with an average between 0.292 and 1.056 100,000, is depicted in blue. A high incidence (greater than the 75th percentile), 1.056 and higher, is shown in dark blue.

Five regions had high mean annual notification rates per 100,000 people: Zakarpattia (3.42), Ivano-Frankivsk (1.48), Mykolayiv (1.34), Kherson (1.29), and Khmelnytskyi (1.2). The regions with the lowest rates were Kharkiv (0.04), Zaporizhzhia (0.15), Zhytomyr (0.21), Dnipropetrovsk (0.22), and Sumy (0.28) (Figures 2, 3).

### 3.3 Age

**Figure 4:**
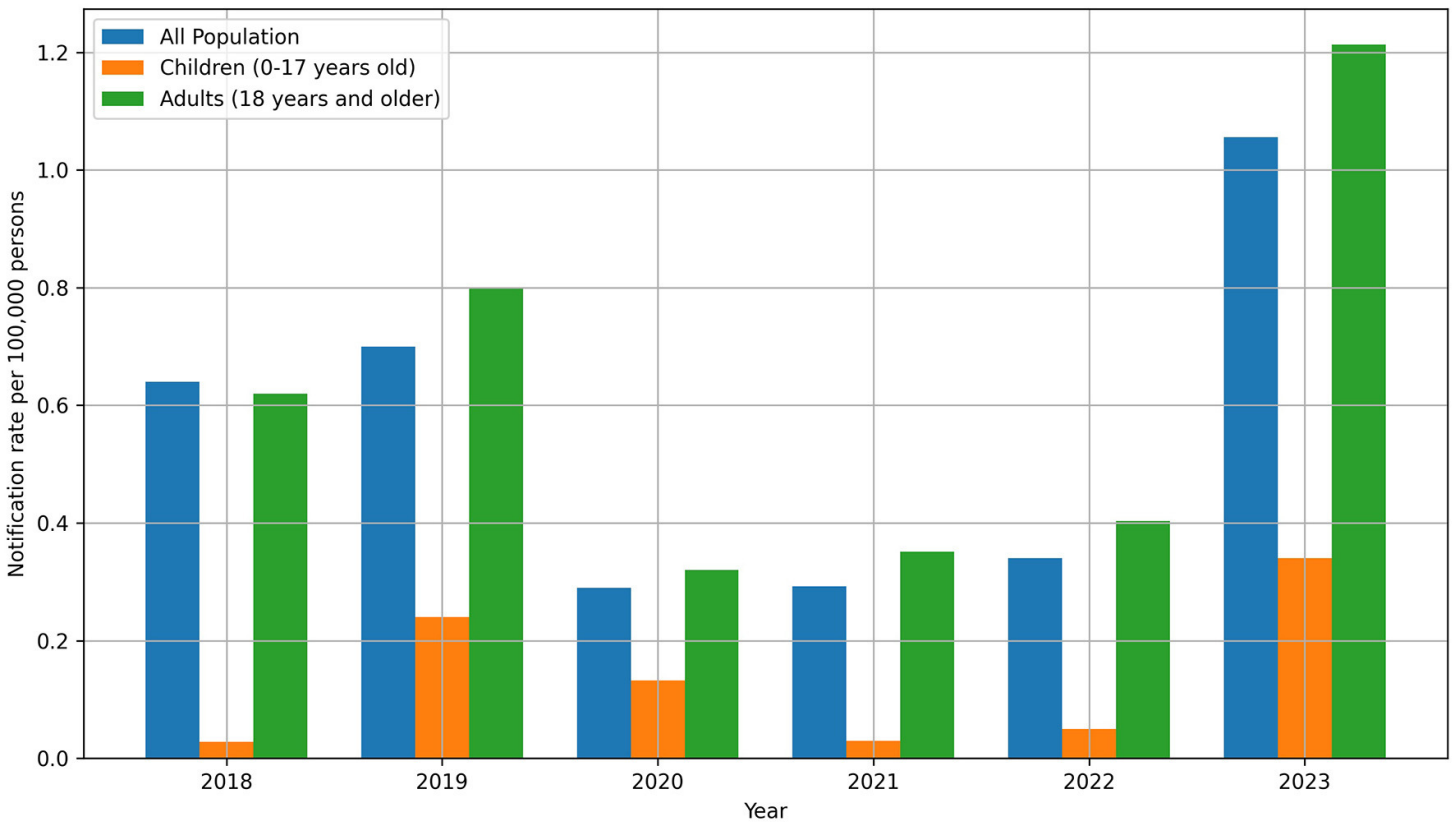
Leptospirosis notification rate in the context of age in the period 2018–2023. In general, in 2023, there was an increase in morbidity both overall (1.056 per 100,000) and among children (0.34 per 100,000).

The analysis revealed a consistent trend of leptospirosis predominantly affecting adults. In 2023, out of the 433 total cases, 408 were adults, and 25 were children. This pattern persisted over the years, with children accounting for 4.4% of cases in 2018, 6.1% in 2019, 8.3% in 2020, 1.6% in 2021, 2.8% in 2022, and 5.8% in 2023, while adults represented the majority of cases in each year. The highest number of children with leptospirosis was reported in Kirovohrad (now Kropyvnytskyi), with 4 children (2.53 per 100,000 persons), and in Zakarpattia, with 6 children (2.08 per 100,000 persons) (Figure 4, Supplementary Tables S3, S4).

## 4 Discussion

Our analysis revealed a significant increase in the notification rate of leptospirosis in Ukraine in 2023. This surge is primarily attributed to the increasing incidence of leptospirosis in Zakarpattia, accounting for 150 of the total 433 cases in Ukraine, and in the Ivano-Frankivsk region, accounting for 34 cases.

Zakarpattia Oblast, located on the western border of Ukraine, shares boundaries with Romania, Hungary, Poland, and Slovakia while also bordering Ivano-Frankivsk and L'viv Oblasts to the east (16). Zakarpattia has several environmental drivers of disease that can influence the prevalence of illnesses such as leptospirosis and is characterized by a temperate continental climate characterized by abundant moisture, moderately warm summers, and mild winters (17). The area sustains an above-average human population density, with 63% residing in rural regions (18). Elevated cases of leptospirosis are frequently documented in locations with plentiful surface freshwater. Zakarpattia's dense river system, influenced by high humidity and mountainous terrains, experiences fluctuations in water levels, spring flooding, and occasional disasters (19). For example, in 2010, prolonged heavy rain during June and July caused flooding that inundated villages in the lowland districts of Zakarpattia (17).

When compared with estimates of countries in the EU and European Economic Area in the period 2010 to 2021, Ukraine's leptospirosis notification rates were considerably higher. For instance, Slovenia and France had the highest total notification rates of 0.82 and 0.76 per 100,000 population, respectively, both substantially lower than those we determined for Ukraine (6). While many European countries witnessed a decline in leptospirosis notifications in the latter half of the 20^th^ century due to reduced agricultural workers and improved living standards, recent trends are less straightforward (6, 20–22). The risk of leptospirosis varies based on environmental and behavioral factors, with heavy rainfall and flooding posing greater risks in tropical countries and recreational water activities linked to the disease in high-income nations. Studies suggest that temperature and rainfall are significantly correlated with leptospirosis incidence, and climate change-related hazards such as human displacement and impaired sewage systems may increase this risk (23).

The prevalence of leptospirosis is notably higher in rural areas globally, where a substantial number of animals are kept (24). Toward the end of the previous century, two zones with a high incidence of leptospirosis in humans emerged in Ukraine (25). The first zone covered the Zakarpattia, Chernivtsi, Ivano-Frankivsk, Ternopil, and Khmelnytskyi regions, while the second extended along the Dnipro River. The complexity of controlling leptospirosis lies in the epizootic and epidemiological features of the disease. Pathogens can establish symbiotic relationships with host animals, persisting in the kidneys without causing active disease. Additionally, wild animals serve as active reservoirs and sources of pathogens for farm animals.

A comprehensive study by Markovych et al. revealed that Icterohaemorrhagiae, Pomona, and Grippotyphosa were the most prevalent serogroups in small mammals. Additionally, Icterohaemorrhagiae, Hebdomadis, and Grippotyphosa were the most common serogroups in humans between 2005 and 2015 (17). It is the view of the authors that these same serogroups might remain predominant in our study period. However, given the dynamic nature of the epidemiological landscape, a detailed report of circulating serovars from 2015 in Ukraine is warranted.

Regarding the lower number of cases observed from 2020 to 2022, it is possible that factors such as reduced testing or reporting due to influences like the COVID-19 pandemic or disruption caused by the war may have contributed. These external factors could have impacted healthcare infrastructure, resource allocation, and surveillance systems, potentially leading to underreporting or underdiagnosis of leptospirosis cases during this period.

## 5 Conclusion

In summary, this study revealed a notable increase in the incidence of leptospirosis in Ukraine from 2018 to 2023, with significant peak in 2023, particularly in the Zakarpattia region. This surge is likely due to environmental factors such as high humidity and flooding. The increase in incidence among children is also concerning, emphasizing the need for targeted interventions. To mitigate the increasing incidence of leptospirosis in Ukraine, it is crucial to implement a comprehensive approach that includes several key strategies. First, increasing public education and awareness is essential. This involves disseminating information to high-risk groups and military personnel about leptospirosis, focusing on risk factors, prevention methods, and early symptoms. Special attention should be given to those in regions with elevated environmental risks and military zones where exposure is heightened. Moreover, the feasibility of vaccination and chemoprophylaxis should be evaluated, particularly for those in high-risk regions or occupational groups such as farmers, sanitation workers, and military personnel. Personal protective measures are also critical; encouraging the use of protective clothing, boots, and gloves can significantly reduce exposure risks. Strengthening the leptospirosis surveillance system will ensure that cases are reported promptly, enabling health authorities to respond quickly to outbreaks. This would require improved tracking of cases, leading to better data collection for future analysis and planning. Environmental management plays a crucial role in controlling leptospirosis, as rodents are a primary reservoir for this disease. Strategies to reduce rodent populations and improve sanitation and sewage systems are necessary to decrease the risk of environmental transmission. Finally, given the potential for increased risk in regions prone to flooding or natural disasters, emergency preparedness plans should be developed. These plans should address potential outbreaks of leptospirosis and other zoonotic diseases, ensuring rapid response and effective containment.

By adopting these strategies, health authorities in Ukraine can significantly reduce the risk of leptospirosis, providing a safer environment for both military and civilian populations.

## Data availability statement

The raw data supporting the conclusions of this article will be made available by the authors, without undue reservation.

## Author contributions

PP: Data curation, Formal analysis, Investigation, Methodology, Visualization, Writing – original draft. VT: Resources, Writing – review & editing. OK: Data curation, Investigation, Project administration, Supervision, Visualization, Writing – original draft.
