# Supplementary material for: Surveillance of human leptospirosis infections in Ukraine between 2018 and 2023
Source: Front Public Health. 2024 Jun 12;12:1394781. doi: 10.3389/fpubh.2024.1394781 (PMC11199720; doi:10.3389/fpubh.2024.1394781)
Supplement: Supplementary file 1 [file Table_1.DOCX]

**Table 1.** Leptospira Reference Strains for the Microscopic Agglutination Test in Ukraine

| **№** | **Serovar** | **Serogroup** | **Strain** |
| --- | --- | --- | --- |
| **1** | Copenhageni | Icterohaemorrhagiae | M 20 |
| **2** | Grippotyphosa type Moskva | Grippotyphosa | Moskva V |
| **3** | Canicola | Canicola | Hond Utrecht IV |
| **4** | Pomona | Pomona | Pomona |
| **5** | Tarassovi | Tarassovi | Perepelitsin |
| **6** | Kabura | Hebdomadis | Kabura |
| **7** | Polonica | Sejroe | 493 Poland |
| **8** | Poi | Javanica | Poi |
| **9** | Autumnalis | Autumnalis | Akiyami A |
| **10** | Bratislava | Australis | Jez Bratislava |
| **11** | Djatzi | Bataviae | HS 26 |
| **12** | Ballum | Ballum | Mus 127 |
| **13** | Pyrogenes | Pyrogenes | Salinem |
| **14** | Cynopteri | Cynopteri | 3522 C |

| **Years** | **2018** | | **2019** | | **2020** | | **2021** | | **2022** | | **2023** | |
| --- | --- | --- | --- | --- | --- | --- | --- | --- | --- | --- | --- | --- |
|  | Total | 100,000 | Total | 100,000 | Total | 100,000 | Total | 100,000 | Total | 100,000 | Total | 100,000 |
| Cherkasy | 5 | 0.41 | 5 | 0.41 | 4 | 0.33 | 3 | 0.25 | 6 | 0.51 | 9 | 0.78 |
| Chernihiv | 6 | 0.59 | 9 | 0.89 | 5 | 0.50 | 0 | 0 | 2 | 0.21 | 24 | 2.52 |
| Chernivtsi | 19 | 2.10 | 13 | 1.44 | 3 | 0.33 | 7 | 0.78 | 2 | 0.22 | 8 | 0.9 |
| Dnipropetrovsk | 4 | 0.12 | 11 | 0.34 | 5 | 0.16 | 3 | 0.09 | 5 | 0.16 | 15 | 0.48 |
| Ivano-Frankivsk | 30 | 2.18 | 29 | 2.11 | 5 | 0.36 | 15 | 1.10 | 8 | 0.59 | 34 | 2.52 |
| Kharkiv | 0 | 0.00 | 3 | 0.11 | 1 | 0.04 | 0 | 0 | 1 | 0.04 | 11 | 0.43 |
| Kherson | 29 | 2.75 | 34 | 3.25 | 8 | 0.77 | 3 | 0.29 | 6 | 0.59 | 4 | 0.40 |
| Khmelnytsk | 17 | 1.33 | 15 | 1.18 | 10 | 0.79 | 4 | 0.32 | 19 | 1.53 | 25 | 2.04 |
| Kirovohrad | 12 | 1.25 | 6 | 0.63 | 5 | 0.53 | 7 | 0.76 | 6 | 0.66 | 21 | 2.34 |
| Kyiv | 9 | 0.31 | 7 | 0.24 | 9 | 0.31 | 5 | 0.17 | 5 | 0.17 | 5 | 0.17 |
| Kyivska | 8 | 0.46 | 9 | 0.51 | 2 | 0.11 | 4 | 0.23 | 8 | 0.45 | 24 | 1.34 |
| Lviv | 24 | 0.95 | 35 | 1.39 | 5 | 0.20 | 11 | 0.44 | 31 | 1.25 | 28 | 1.14 |
| Mykolayiv | 24 | 2.09 | 15 | 1.32 | 10 | 0.88 | 20 | 1.79 | 4 | 0.36 | 14 | 1.28 |
| Odesa | 5 | 0.21 | 16 | 0.67 | 4 | 0.17 | 3 | 0.13 | 3 | 0.13 | 9 | 0.38 |
| Poltava | 3 | 0.21 | 9 | 0.64 | 2 | 0.14 | 2 | 0.15 | 5 | 0.37 | 3 | 0.22 |
| Rivne | 2 | 0.17 | 7 | 0.60 | 1 | 0.09 | 2 | 0.17 | 2 | 0.17 | 9 | 0.79 |
| Sumy | 5 | 0.45 | 6 | 0.55 | 1 | 0.09 | 2 | 0.19 | 2 | 0.19 | 2 | 0.19 |
| Ternopil | 16 | 1.52 | 16 | 1.53 | 5 | 0.48 | 7 | 0.68 | 5 | 0.49 | 15 | 1.47 |
| Vinnytsia | 17 | 1.07 | 18 | 1.15 | 10 | 0.64 | 4 | 0.26 | 9 | 0.59 | 11 | 0.73 |
| Volyn | 3 | 0.29 | 3 | 0.29 | 1 | 0.10 | 1 | 0.10 | 2 | 0.20 | 8 | 0.79 |
| Zakarpattia | 33 | 2.63 | 23 | 1.83 | 22 | 1.75 | 19 | 1.52 | 9 | 0.72 | 150 | 12.08 |
| Zaporizhzhia | 1 | 0.06 | 0 | 0.00 | 0 | 0.00 | 0 | 0.00 | 0 | 0.00 | 1 | 0.06 |
| Zhytomyr | 1 | 0.08 | 6 | 0.49 | 2 | 0.16 | 0 | 0.00 | 1 | 0.08 | 3 | 0.25 |
| Ukraine (total) | 273 | 0.64 | 295 | 0.70 | 120 | 0.29 | 122 | 0.29 | 141 | 0.34 | 433 | 1.05 |

**Table 2.** Notification Rates in Ukraine 2018-2023 (absolute values and notification rates per 100,000 persons)

| **Years** | **2018** | | | **2019** | | | **2020** | | | **2021** | | | **2022** | | | **2023** | | |
| --- | --- | --- | --- | --- | --- | --- | --- | --- | --- | --- | --- | --- | --- | --- | --- | --- | --- | --- |
|  | All population | Children | Adult | All population | Children | Adult | All population | Children | Adult | All population | Children | Adult | All population | Children | Adult | All population | Children | Adult |
| Cherkasy | 5 | 0 | 5 | 5 | 1 | 4 | 4 | 0 | 4 | 3 | 0 | 3 | 6 | 0 | 6 | 9 | 0 | 9 |
| Chernihiv | 6 | 0 | 6 | 9 | 1 | 8 | 5 | 0 | 5 | 0 | 0 | 0 | 2 | 0 | 2 | 24 | 2 | 22 |
| Chernivtsi | 19 | 0 | 19 | 13 | 0 | 13 | 3 | 0 | 3 | 7 | 0 | 7 | 2 | 0 | 2 | 8 | 1 | 7 |
| Dnipropetrovsk | 4 | 0 | 4 | 11 | 0 | 11 | 5 | 0 | 5 | 3 | 0 | 3 | 5 | 0 | 5 | 15 | 0 | 15 |
| Ivano-Frankivsk | 30 | 1 | 29 | 29 | 1 | 28 | 5 | 0 | 5 | 15 | 0 | 15 | 8 | 0 | 8 | 34 | 1 | 33 |
| Kharkiv | 0 | 0 | 0 | 3 | 0 | 3 | 1 | 0 | 1 |  | 0 | 0 | 1 | 0 | 1 | 11 | 0 | 11 |
| Kherson | 29 | 2 | 27 | 34 | 0 | 34 | 8 | 1 | 7 | 3 | 0 | 3 | 6 | 0 | 6 | 4 | 0 | 4 |
| Khmelnytsk | 17 | 1 | 16 | 15 | 1 | 14 | 10 | 0 | 10 | 4 | 0 | 4 | 19 | 1 | 18 | 25 | 3 | 22 |
| Kirovohrad | 12 | 2 | 10 | 6 |  | 6 | 5 | 1 | 4 | 7 | 1 | 6 | 6 | 0 | 6 | 21 | 4 | 17 |
| Kyiv | 9 | 1 | 8 | 7 | 1 | 6 | 9 | 0 | 9 | 5 | 1 | 4 | 5 | 0 | 5 | 5 | 0 | 5 |
| Kyivska oblast | 8 | 0 | 8 | 9 | 0 | 9 | 2 | 0 | 2 | 4 | 0 | 4 | 8 | 1 | 7 | 24 | 1 | 23 |
| Lviv | 24 | 0 | 24 | 35 | 0 | 35 | 5 | 0 | 5 | 11 | 0 | 11 | 31 | 0 | 31 | 28 | 0 | 28 |
| Mykolayiv | 24 | 1 | 23 | 15 | 2 | 13 | 10 | 3 | 7 | 20 | 0 | 20 | 4 | 0 | 4 | 14 | 2 | 12 |
| Odesa | 5 | 0 | 5 | 16 | 3 | 13 | 4 | 2 | 2 | 3 | 0 | 3 | 3 | 0 | 3 | 9 | 0 | 9 |
| Poltava | 3 | 0 | 3 | 9 | 0 | 9 | 2 | 1 | 1 | 2 | 0 | 2 | 5 | 1 | 4 | 3 | 0 | 3 |
| Rivne | 2 | 0 | 2 | 7 | 1 | 6 | 1 | 0 | 1 | 2 | 0 | 2 | 2 | 0 | 2 | 9 | 1 | 8 |
| Sumy | 5 | 0 | 5 | 6 | 1 | 5 | 1 | 0 | 1 | 2 | 0 | 2 | 2 | 0 | 2 | 2 | 0 | 2 |
| Ternopil | 16 | 2 | 14 | 16 | 1 | 15 | 5 | 1 | 4 | 7 | 0 | 7 | 5 | 0 | 5 | 15 | 1 | 14 |
| Vinnytsia | 17 | 2 | 15 | 18 | 3 | 15 | 10 | 1 | 9 | 4 | 0 | 4 | 9 | 0 | 9 | 11 | 3 | 8 |
| Volyn | 3 | 0 | 3 | 3 | 1 | 2 | 1 | 0 | 1 | 1 | 0 | 1 | 2 | 0 | 2 | 8 | 0 | 8 |
| Zakarpattia | 33 | 0 | 33 | 23 | 1 | 22 | 22 | 0 | 22 | 19 | 0 | 19 | 9 | 0 | 9 | 150 | 6 | 144 |
| Zaporizhzhia | 1 | 0 | 1 | 0 | 0 | 0 | 0 | 0 | 0 | 0 | 0 | 0 | 0 | 0 | 0 | 1 | 0 | 1 |
| Zhytomyr | 1 | 0 | 1 | 6 | 0 | 6 | 2 | 0 | 2 | 0 | 0 | 0 | 1 | 1 | 0 | 3 | 0 | 3 |
| Ukraine (total) | 273 | 12 | 261 | 295 | 18 | 277 | 120 | 10 | 110 | 122 | 2 | 120 | 141 | 4 | 137 | 433 | 25 | 408 |

**Table 3.** Leptospirosis Cases by Age Group in the Period 2018-2023 (absolute values)

| **Years** | **2018** | | | **2019** | | | **2020** | | | **2021** | | | **2022** | | | **2023** | | | |
| --- | --- | --- | --- | --- | --- | --- | --- | --- | --- | --- | --- | --- | --- | --- | --- | --- | --- | --- | --- |
|  | All population | Children | Adult | All population | Children | Adult | All population | Children | Adult | All population | Children | Adult | All population | Children | Adult | All population | Children | Adult |  |
| Cherkasy | 0.41 | 0.00 | 0.49 | 0.41 | 0.50 | 0.39 | 0.33 | 0.00 | 0.40 | 0.25 | 0.00 | 0.30 | 0.51 | 0.00 | 0.61 | 0.78 | 0.00 | 0.93 |  |
| Chernihiv | 0.59 | 0.00 | 0.70 | 0.89 | 0.61 | 0.94 | 0.50 | 0.00 | 0.60 | 0.00 | 0.00 | 0.00 | 0.21 | 0.00 | 0.25 | 2.52 | 1.32 | 2.75 |  |
| Chernivtsi | 2.10 | 0.00 | 2.64 | 1.44 | 0.00 | 1.81 | 0.33 | 0.00 | 0.42 | 0.78 | 0.00 | 0.98 | 0.22 | 0.00 | 0.28 | 0.90 | 0.55 | 0.99 |  |
| Dnipropetrovsk | 0.12 | 0.00 | 0.15 | 0.34 | 0.00 | 0.42 | 0.16 | 0.00 | 0.19 | 0.09 | 0.00 | 0.12 | 0.16 | 0.00 | 0.19 | 0.48 | 0.00 | 0.59 |  |
| Ivano-Frankivsk | 2.18 | 0.36 | 2.64 | 2.11 | 0.36 | 2.55 | 0.36 | 0.00 | 0.46 | 1.10 | 0.00 | 1.38 | 0.59 | 0.00 | 0.74 | 2.52 | 0.37 | 3.06 |  |
| Kharkiv | 0.00 | 0.00 | 0.00 | 0.11 | 0.00 | 0.13 | 0.04 | 0.00 | 0.04 | 0.00 | 0.00 | 0.00 | 0.04 | 0.00 | 0.05 | 0.43 | 0.00 | 0.51 |  |
| Kherson | 2.75 | 1.01 | 3.15 | 3.25 | 0.00 | 4.01 | 0.77 | 0.51 | 0.83 | 0.29 | 0.00 | 0.36 | 0.59 | 0.00 | 0.73 | 0.4 | 0.00 | 0.49 |  |
| Khmelnytsk | 1.33 | 0.42 | 1.53 | 1.18 | 0.42 | 1.35 | 0.79 | 0.00 | 0.97 | 0.32 | 0.00 | 0.39 | 1.53 | 0.43 | 1.78 | 2.04 | 1.32 | 2.20 |  |
| Kirovohrad | 1.25 | 1.17 | 1.27 | 0.63 | 0.00 | 0.77 | 0.53 | 0.60 | 0.52 | 0.76 | 0.61 | 0.79 | 0.66 | 0.00 | 0.8 | 2.34 | 2.53 | 2.30 |  |
| Kyiv | 0.31 | 0.19 | 0.34 | 0.24 | 0.18 | 0.26 | 0.31 | 0.00 | 0.38 | 0.17 | 0.18 | 0.17 | 0.17 | 0.00 | 0.21 | 0.17 | 0.00 | 0.21 |  |
| Kyivska oblast | 0.46 | 0.00 | 0.57 | 0.51 | 0.00 | 0.64 | 0.11 | 0.00 | 0.14 | 0.23 | 0.00 | 0.28 | 0.45 | 0.28 | 0.49 | 1.34 | 0.27 | 1.62 |  |
| Lviv | 0.95 | 0.00 | 1.18 | 1.39 | 0.00 | 1.73 | 0.20 | 0.00 | 0.25 | 0.44 | 0.00 | 0.55 | 1.25 | 0.00 | 1.55 | 1.14 | 0.00 | 1.41 |  |
| Mykolayiv | 2.09 | 0.48 | 2.44 | 1.32 | 0.96 | 1.39 | 0.88 | 1.45 | 0.76 | 1.79 | 0.00 | 2.19 | 0.36 | 0.00 | 0.44 | 1.28 | 1.01 | 1.34 |  |
| Odesa | 0.21 | 0.00 | 0.26 | 0.67 | 0.65 | 0.68 | 0.17 | 0.43 | 0.11 | 0.13 | 0.00 | 0.16 | 0.13 | 0.00 | 0.16 | 0.38 | 0.00 | 0.48 |  |
| Poltava | 0.21 | 0.00 | 0.25 | 0.64 | 0.00 | 0.77 | 0.14 | 0.43 | 0.09 | 0.15 | 0.00 | 0.17 | 0.37 | 0.44 | 0.35 | 0.22 | 0.00 | 0.27 |  |
| Rivne | 0.17 | 0.00 | 0.23 | 0.6 | 0.36 | 0.68 | 0.09 | 0.00 | 0.11 | 0.17 | 0.00 | 0.23 | 0.17 | 0.00 | 0.23 | 0.79 | 0.37 | 0.92 |  |
| Sumy | 0.45 | 0.00 | 0.54 | 0.55 | 0.58 | 0.54 | 0.09 | 0.00 | 0.11 | 0.19 | 0.00 | 0.22 | 0.19 | 0.00 | 0.23 | 0.19 | 0.00 | 0.23 |  |
| Ternopil | 1.52 | 1.00 | 1.63 | 1.53 | 0.51 | 1.76 | 0.48 | 0.51 | 0.47 | 0.68 | 0.00 | 0.83 | 0.49 | 0.00 | 0.60 | 1.47 | 0.53 | 1.69 |  |
| Vinnytsia | 1.07 | 0.69 | 1.16 | 1.15 | 1.04 | 1.17 | 0.64 | 0.35 | 0.71 | 0.26 | 0.00 | 0.32 | 0.59 | 0.00 | 0.72 | 0.73 | 1.09 | 0.65 |  |
| Volyn | 0.29 | 0.00 | 0.38 | 0.29 | 0.42 | 0.25 | 0.10 | 0.00 | 0.13 | 0.10 | 0.00 | 0.13 | 0.20 | 0.00 | 0.25 | 0.79 | 0.00 | 1.02 |  |
| Zakarpattia | 2.63 | 0.00 | 3.42 | 1.83 | 0.34 | 2.28 | 1.75 | 0.00 | 2.29 | 1.52 | 0.00 | 1.98 | 0.72 | 0.00 | 0.94 | 12.08 | 2.08 | 15.11 |  |
| Zaporizhzhia | 0.06 | 0.00 | 0.07 | 0.00 | 0.00 | 0.00 | 0.00 | 0.00 | 0.00 | 0.00 | 0.00 | 0.00 | 0.00 | 0.00 | 0.00 | 0.06 | 0.00 | 0.07 |  |
| Zhytomyr | 0.08 | 0.00 | 0.10 | 0.49 | 0.00 | 0.61 | 0.16 | 0.00 | 0.2 | 0.00 | 0.00 | 0.00 | 0.08 | 0.43 | 0.00 | 0.25 | 0.00 | 0.32 |  |
| Ukraine (total) | 0.64 | 0.028 | 0.62 | 0.7 | 0.24 | 0.8 | 0.29 | 0.13 | 0.32 | 0.29 | 0.03 | 0.35 | 0.34 | 0.05 | 0.40 | 1.05 | 0.34 | 1.21 |  |

**Table 4.** Leptospirosis Notification Rate in the Context of Age in the Period 2018-2023 (absolute values and notification rates per 100,000 persons)
